# Liposomal delivery of ferritin heavy chain 1 (FTH1) siRNA in patient xenograft derived glioblastoma initiating cells suggests different sensitivities to radiation and distinct survival mechanisms

**DOI:** 10.1371/journal.pone.0221952

**Published:** 2019-09-06

**Authors:** Vagisha Ravi, Achuthamangalam B. Madhankumar, Thomas Abraham, Becky Slagle-Webb, James R. Connor

**Affiliations:** 1 Department of Neurosurgery, Penn State College of Medicine, Hershey, Pennsylvania, United States of America; 2 Department of Neural and Behavioral Sciences, Penn State College of Medicine, Hershey, Pennsylvania, United States of America; Sechenov First Medical University, RUSSIAN FEDERATION

## Abstract

Elevated expression of the iron regulatory protein, ferritin heavy chain 1 (FTH1), is increasingly being associated with high tumor grade and poor survival outcomes in glioblastoma. Glioma initiating cells (GICs), a small population of stem-like cells implicated in therapeutic resistance and glioblastoma recurrence, have recently been shown to exhibit increased FTH1 expression. We previously demonstrated that FTH1 knockdown enhanced therapeutic sensitivity in an astrocytoma cell line. Therefore, in this study we developed a liposomal formulation to enable the *in vitro* delivery of FTH1 siRNA in patient xenograft derived GICs from glioblastomas with pro-neural and mesenchymal transcriptional signatures to interrogate the effect of FTH1 downregulation on their radiation sensitivity. Transfection with siRNA decreased FTH1 expression significantly in both GICs. However, there were inherent differences in transfectability between pro-neural and mesenchymal tumor derived GICs, leading us to modify siRNA: liposome ratios for comparable transfection. Moreover, loss of FTH1 expression resulted in increased extracellular lactate dehydrogenase activity, executioner caspase 3/7 induction, substantial mitochondrial damage, diminished mitochondrial mass and reduced cell viability. However, only GICs from pro-neural glioblastoma showed marked increase in radiosensitivity upon FTH1 downregulation demonstrated by decreased cell viability, impaired DNA repair and reduced colony formation subsequent to radiation. In addition, the stemness marker Nestin was downregulated upon FTH1 silencing only in GICs of pro-neural but not mesenchymal origin. Using liposomes as a siRNA delivery system, we established FTH1 as a critical factor for survival in both GIC subtypes as well as a regulator of radioresistance and stemness in pro-neural tumor derived GICs. Our study provides further evidence to support the role of FTH1 as a promising target in glioblastoma.

## Introduction

Glioblastoma continues to remain the most common and refractory solid brain tumor. Despite maximal standard treatment [1] consisting of surgical resection followed by radiation and chemotherapy, there is an invariable and nearly universal recurrence attributed to the presence of glioblastoma initiating cells (GICs) [2, 3]. GICs are stem-like cells characterized by surface expression of CD133 (prominin), high tumorigenic potential *in vivo* and increased capacity for angiogenesis [4, 5], invasion [6] and immune system evasion [7, 8] among others. Yet it is their efficient drug efflux [9, 10] and DNA repair capabilities [2, 11] that makes GICs significantly more resistant than their non-stem counterparts [2, 12], allowing them to circumvent treatment and repopulate the tumor [13].

A prominent cytoprotective protein, ferritin, is correlated with higher tumor grade and poor prognosis in glioblastoma [14]. Ferritin forms a nanocage comprising 24 subunits of ferritin heavy chain (FTH1) and ferritin light chain (FTL) peptides in differing ratios [15]. FTL functions mainly to nucleate oxidized iron and has recently been found to contribute to glioblastoma cell proliferation through regulation of GADD45/JNK pathway [16]. FTH1, in addition to nucleation, possesses ferroxidase activity which limits iron for the Fenton reaction and protects the cell against oxidative stress. In addition to residing within the cytosol, ferritin can traverse into the nucleus but only FTH1 can interact with DNA [17, 18] where it has been reported to protect corneal epithelial cells from UV radiation [19] and the DNA of some cancer cells from oxidative damage [19, 20]. We have previously shown that decreasing FTH1 sensitizes glioma cells to the chemotherapy with BCNU and radiation [21]. Additionally, Schonberg et al recently reported that the expression of FTH1 and ferritin light chain (FTL) is elevated in the CD133+ over CD133- fraction in GICs and that downregulation of both subunits with shRNA led to complete loss of tumorigenicity *in vivo* [14].

Transcriptional profiling of glioblastoma tumors has shown different subtypes to possess intrinsic differences in radiation responses [22, 23]. Radiation is the cornerstone of glioblastoma treatment and efficient DNA damage repair in GICs impede effective radiation therapy. We therefore wanted to determine the effect of FTH1 loss on GICs isolated from relatively radio sensitive (proneural, PN) and radio resistant (Mesenchymal, MES) glioblastomas. This study describes the development of a liposomal formulation that enables efficient transfection and downregulation of FTH1 expression *in vitro* and its effects on radiosensitivity of patient derived GICs.

## Materials and methods

### Materials

The lipids DC-Cholesterol/Dioleoyl Phosphatidylethanolamine (DOPE) (30:70, w/w), 1,2-dioleoyl-3-trimethylammonium-propane (DOTAP) and N1-[2-((1S)-1-[(3-aminopropyl)amino]-4-[di(3-amino-propyl) amino] butylcarboxamido) ethyl]-3,4-di [oleyloxy]-benzamide (MVL5) were purchased from Avanti Polar Lipids, Inc. (Alabaster, AL). Patient tumor derived cells generated as described in [14] were a gift from Dr. Jeremy Rich at University of California, San Diego. FTH1 siRNA (Santa Cruz, Cat# 40575) and control silencer^™^ firefly luciferase siRNA (Thermo Fisher, Cat #AM4629) were used for transfection. Antibodies used were as follows. FTH1 (Cell Signaling, Cat #4393, 1:500), phosphoϒH2AX (Cell Signaling Cat #9718, 1:500 western blot; 1:200 immunocytochemistry), β-actin (Sigma, Cat #A5411, 1:5000), EEA1 (Santa Cruz Cat# 137130, 1:200), TOMM20 (Santa Cruz Cat#17764 1:100 western blot, 1:250 immunocytochemistry) and Nestin (Abcam, Cat # 22035, 1:500). HRP-conjugated secondary antibodies for western blot were purchased from GE Healthcare. DiI, siGLO and Alexa Fluor antibodies were purchased from ThermoFisher. MTS (Cat#G3582) and Caspase-Glo® 3/7 assay (G8090) were purchased from Promega and LDH assay kit (Cat# 11644793001) from Sigma.

### Preparation and characterization of liposomes

DC-Cholesterol/DOPE, DOTAP and MVL5 (1:1:0.2) with or without membrane stain, DiI (ThermoFisher), were used to form a lipid film, hydrated with 1X phosphate buffered saline (PBS), sonicated and passed through a 0.1μm polycarbonate membrane followed by 0.05μm polycarbonate membrane, five times each, at 37° C using a nitrogen pressure operated Lipex extruder (Northern Lipids, Inc.)[24]. To prepare liposomes labeled with DiI, 1mg/ml DiI solution in chloroform was added to the lipid mixture and processed as described previously. The liposomes were then concentrated by passing first through a Sephadex G-25 medium (GE Life Sciences) column followed by concentration at 4°C in a centriprep30k concentrator (ThermoFisher). Liposomes were stored at 4°C for upto 4 weeks. Particle size, polydispersity index (PDI) and zeta potential analysis were performed using the PALS Zeta Potential Analyzer (Ver. 3.16; Brookhaven Instruments Corp.).

### Cell culture and transfection

Tumor tissue from patients with primary glioblastoma was used to isolate patient xenograft derived (PDX) CD133+ T3691 and T387 GICs. PDX GICs were cultured as neurospheres in neurobasal media without phenol red (Gibco) supplemented with 2% B27 without vitamin A (v/v) (Gibco), 1% sodium pyruvate (v/v) (Gibco), 1% GlutaMax^™^ (v/v) (Gibco) and 20ng/ml of recombinant human epidermal growth factor (EGF) and 4ng/ml of fibroblast growth factor (FGF) (R & D systems) at 37°C and 5%CO_2_ [14]. Both cell lines were maintained at low passages (passage 3–7) and a comparative analysis of early and late passage cells did not show significant changes in STR profile. For transfection, neurospheres were dissociated by incubating in Accutase® (Innovative cell technologies) at 37°C. The cells were then washed and re-suspended in neurobasal media and viability counts were obtained by trypan blue staining. 1x10^5^ cells/ml of viable cells were plated for 72 hours on 6-well plates pre-coated for an hour with Geltrex^TM^ (1.6μl/ml) (ThermoFisher) and cultured as adherent monolayers. When cells were 70% confluent, they were transfected by addition of 2μg of FTH1 siRNA (Santa Cruz) or control silencer firefly luciferase siRNA (ThermoFisher) complexed with liposomes in a 2μg:4 μl ratio (siRNA: liposome) for T3691 cells and 2μg:8μl ratio for T387 cells. Cells were incubated with complexes for the duration of the experiment. Control cells were treated with a volume of liposomes corresponding to that in complexes or left untreated.

### Western blot

Cells were lysed in RIPA buffer with protease inhibitors. 10–20μg of total protein was run on 4–20% TGX gradient SDS gel (BioRad) as previously described [24] and probed with antibodies specified in the materials section. Densitometry was performed using Image studio lite v5.2 (Licor). Expression levels were normalized to β-actin. Experiments were performed in triplicate.

### Immunocytochemistry

Cells were fixed with 4% paraformaldehyde, incubated with primary antibody overnight followed by secondary antibody for an hour and mounted with Antifade-DAPI (ThermoFisher). GICs were transfected with siGLO green/DiI-liposome complexes for 24h, fixed and mounted. For endosomal uptake study, cells were transfected with control siRNA-DiI-liposomes for 2h at 37°C and stained as described in [21] with early endosomal antigen (EEA1) antibody. For mitochondrial damage assessment, cells were transfected for 24h and immunostained with TOMM20. For quantification of Nestin positive cells, GICs were transfected for 48h and stained with Nestin antibody. Three fields were imaged per condition and number of Nestin positive cells quantified with Image J version 1.49. Results were confirmed by an individual blinded to the identity of the treatment groups. Leica SP8 scanning confocal microscope and Adobe Photoshop CC 2018 were used for image acquisition and processing.

### Cytotoxicity analysis

LDH levels in media from transfected cells was measured as per manufacturer’s instructions. For viability assay, cells (1x10^4^) were transfected for 36 hours in 96 well plates and exposed to ϒ-radiation at 0Gy, 4Gy or 8Gy and their viability measured using MTS assay, 24 and 48h after radiation exposure. Caspase 3/7 activity was measured at 48h post knockdown as per manufacturer’s instructions.

### Quantification of phospho- H2AX foci

GICs were transfected for 24h, then radiated at 2Gy or 0Gy, fixed and stained with anti-pϒH2AX antibody. Five fields per condition were imaged and total foci and number of nuclei in each field was quantified using an automated program in Volocity®. Briefly, average size of nuclei and foci was pre-defined. Following the removal of visually apoptotic nuclei, the images were batch processed and data exported to Microsoft^®^ Excel. Total number of foci from five fields was normalized to number of nuclei per field. Counts were normalized to number of foci/nucleus in untransfected controls.

### Colony formation assay

Cells were transfected for 12 hours in 6 well plates, dissociated with Accutase, washed, counted and seeded in Geltrex® coated 6 well plates at 2.5 x10^3^ cells (T387) or 5 x10^3^ cells (T3691) per well. After allowing attachment for another 12 hours, cells were irradiated at 2Gy or 0Gy. Media was changed 24 hours after radiation. 18 days later, colonies were fixed with 100% methanol, stained with 0.5% (w/v) crystal violet and counted under a microscope. Results were verified by an individual blinded to the identity of the treatment groups.

### Statistical analysis

One-way ANOVA with Tukey’s post hoc or two-Way ANOVA with Bonferroni post hoc correction was used to evaluate differences between multiple groups where appropriate with GraphPad Prism 4.0 (GraphPad Software Inc., San Diego, CA). Significance was set at p <0.05.

## Results

### Liposomes efficiently transfect PDX GICs *in vitro*

**Fig 1:**
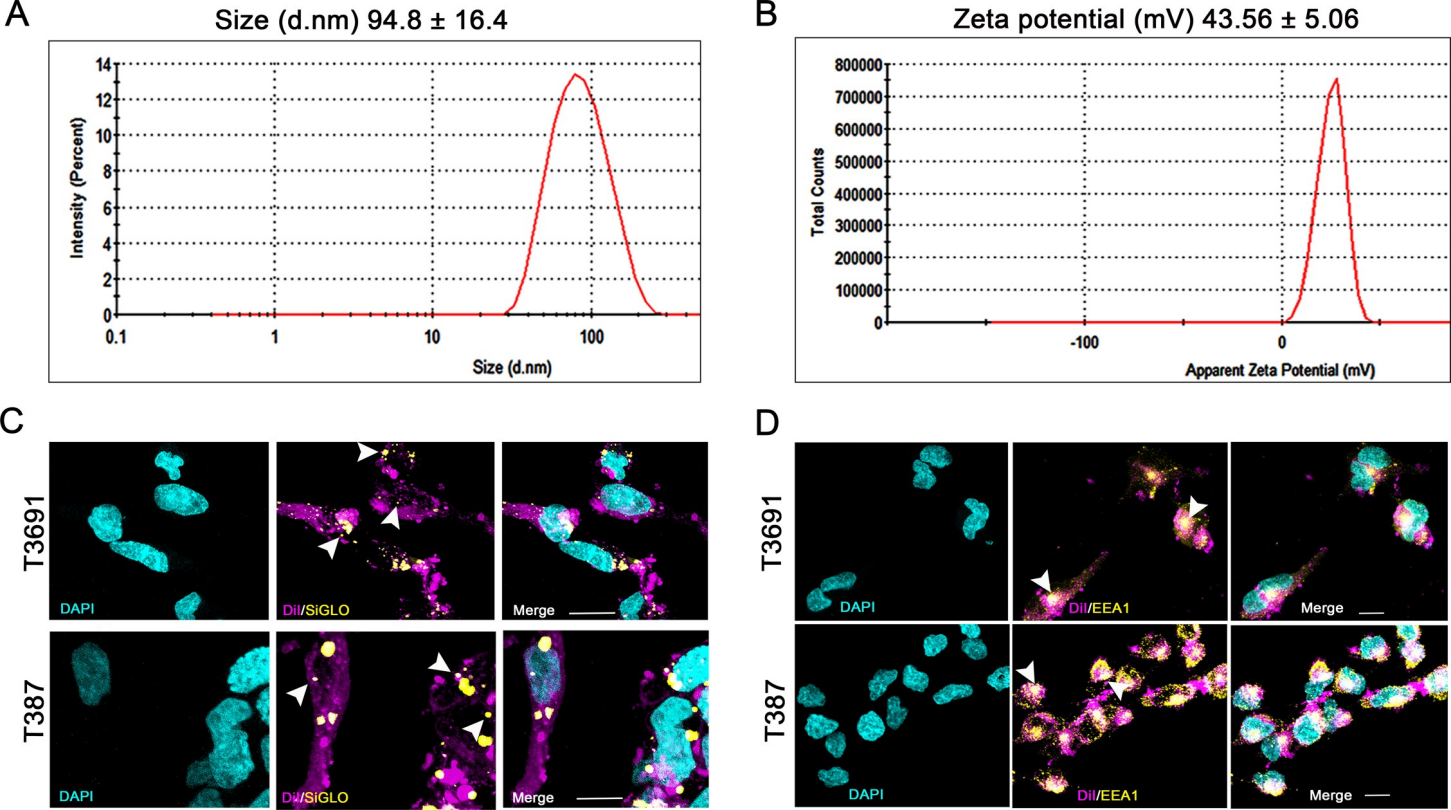
Liposome characterization. (A) Mean (±SD) particle size, polydispersity index and (B) zeta potential of three batches of liposomes was 94.8 ±16.4 nm in diameter (d.nm) and 43.56±5.06 millivolt (mV) respectively. (Table 1) Zeta potential of siRNA (μg): liposome (μl) complexes used for transfection of T3691 (2μg:4μl) and T387 (2μg:8μl) GICs was determined to be anionic and cationic respectively. (C) DiI labeled liposomes (magenta) transfected show colocalization in EEA1 labeled early endosomes (yellow) indicating endosomal uptake mechanism. (D) Transfection with DiI-liposome (magenta): siGLO (yellow) complexes showed efficient uptake and localization in cytosol indicating endosomal escape. Nuclei were counterstained with DAPI (cyan). Arrowheads point to areas of EEA1-DiI colocalization (C) or siGLO foci (D). Scale bar = 10μm.

Gene therapy with siRNA is an attractive therapeutic option that has been successfully used in many preclinical models of cancers including glioma, but siRNA delivery has proven challenging [25]. Although we have previously shown efficacy of FTH1 siRNA in a subcutaneous glioblastoma model using astrocytoma cells [24], we found this liposomal formulation had poor transfection efficiency in GICs. We found that addition of the pentavalent lipid MVL5 resulted in a robust knockdown of FTH1 in GICs when added to our formulation. Liposomes thus generated had an expected particle size, distribution and cationic zeta potential (Fig 1A and 1B). Gel shift assays confirmed efficient complex formation with siRNA indicated by slower migration of siRNA: liposome complexes versus siRNA alone (S1 Fig). Colocalization of DiI-liposomes with EEA1 confirmed endosomal uptake of these complexes (Fig 1D). We then qualitatively determined transfection efficiency and endosomal escape of complexes by transfecting GICs with DiI-liposomes complexed with a fluorescent siRNA, siGLO and observed accumulation of siGLO foci in both T3691 and T387 GICs indicating successful transfection (Fig 1E).

### Transfection of PDX GICs with FTH1 siRNA-liposome complexes results in efficient knockdown

**Fig 2:**
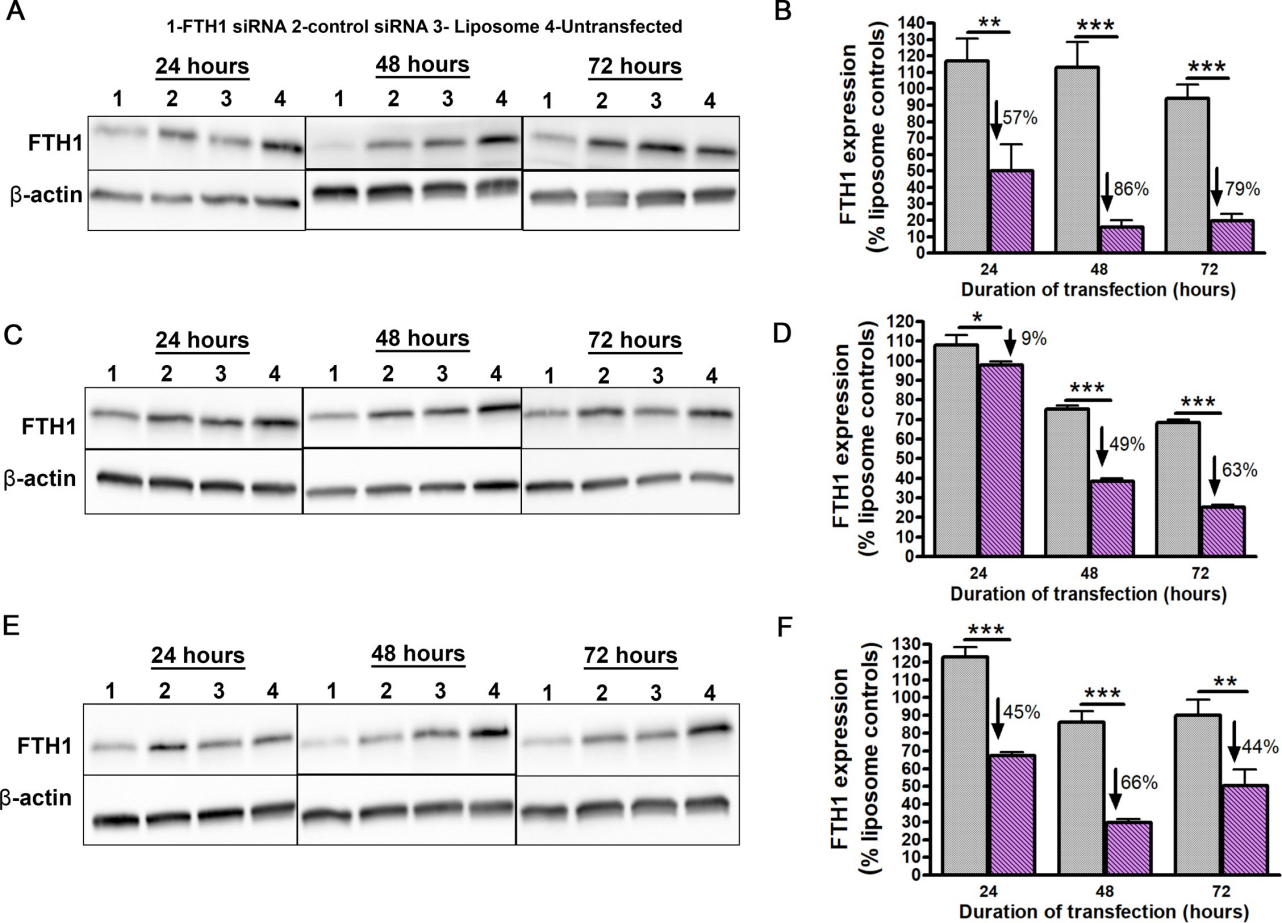
Liposomal delivery of siRNA efficiently downregulates FTH1 expression in GICs. (A) Western blot analysis of FTH1 expression for T3691 cells transfected with siRNA: liposome ratio of 2μg:4μl. (B) Densitometric quantification of T3691 cells in (A) showed significant reduction in FTH1 expression relative to control siRNA treated cells at 24h (57%,p<0.01), 48h (86%, p<0.001) and 72h (79%,p<0.001).(C) Western blot analysis of FTH1 expression in T387 cells transfected at ratios of 2μg:4μl. (D) Densitometric analysis of T387 cells in (C) showed comparatively poor knockdown at 24h (9%, p<0.05) and 48h (49%, p<0.001) with the maximum knockdown achieved at 72h post transfection (63%, p<0.001). (E) Western blot of FTH1 expression in T387 cells transfected at 2μg:8μl. (F) Densitometric analysis of (E) showed that changing siRNA: liposomes ratio to 2:8 in T387 cells improved knockdown significantly at 24h (45%, p<0.001), 48h (66%, p<0.001) and 72h (44%, p<0.01). Expression levels were normalized to endogenous actin levels and then to expression of FTH1 in liposome treated controls. Percentage values are relative to control siRNA from three independent experiments.

We next tested the ability of FTH1 siRNA-liposome complex to downregulate FTH1 expression in GICs. Based on our previous studies [21], we complexed 2μg siRNA with 4μl liposome which enabled efficient transfection of T3691 cells which showed the most significant knockdown at 48h post transfection (Fig 2A and 2B). However, transfecting T387 GICs with these complexes resulted in a lower transfection efficiency (Fig 2C and 2D). Changing the ratio to 2μg:8μl produced cationic complexes capable of achieving comparable knockdown in T387 GICs (Fig 2B and 2D). Since changes in siRNA: lipid ratio can have significant influence on charge and behavior of lipoplexes [26], we analyzed the zeta potential of our complexed particles (Table 1). We discovered that a lower ratio of siRNA: liposome produced cationic lipoplexes which was sufficient and necessary for comparable transfection of T387 cells. Thus, T387 cells were henceforth transfected at the effective ratio of 2μg:8μl to ensure comparable knockdown with T3691 GICs.

**Table 1:** Zeta potential analysis of siRNA: liposome complexes used for GIC transfection.

Sample	siRNA (μg): liposome (μl)	Zeta Potential (mV) (Mean ± SD)
Uncomplexed liposome	-	43.56 ± 5.06
H-ferritin siRNA/liposome complex	2:4	-43.70 ± 3.6
	2:8	28.45 ± 5.85
Control luciferase siRNA/liposome complex	2:4	-64.7 ± 7.4
	2:8	33.95 ± 1.85

### FTH1 regulates cell survival in PDX GICs

**Fig 3:**
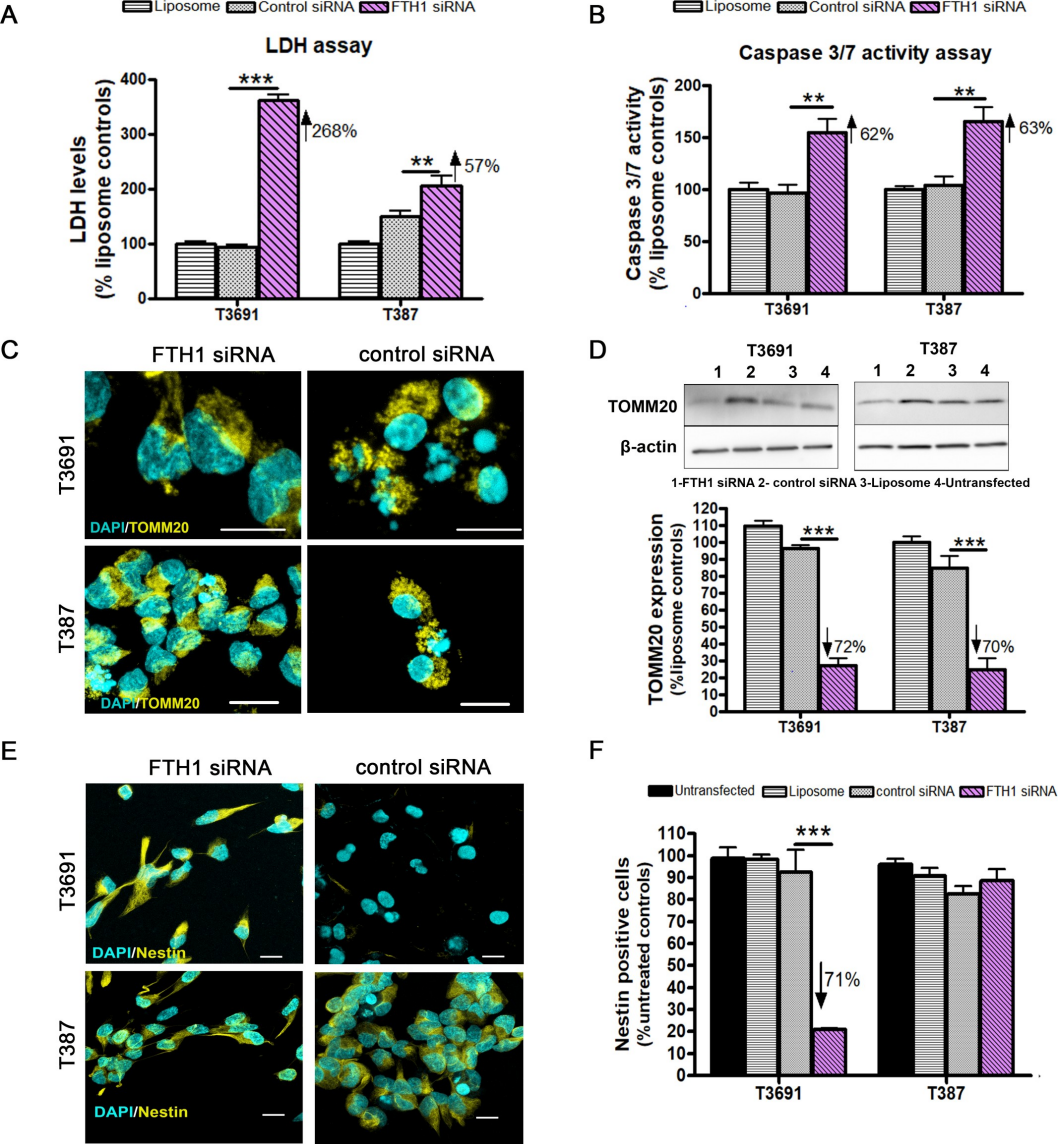
FTH1 knockdown is detrimental to GIC survival. (A) Knockdown of FTH1 led to increased extracellular LDH release in T3691 (268%, p<0.001) and T387 (57%, p<0.01). (B) Executioner caspase 3/7 activity was similarly elevated in T3691 (62%, p<0.01) and T387 cells (63%, p<0.01). (C) Mitochondrial damage was assessed by TOMM20 staining (yellow) at 24h post-transfection with FTH1 siRNA showing perinuclear localization (yellow). Scale bar = 10μm. (D) Decrease in mitochondrial mass, measured by quantifying TOMM20, occurred in both T3691 (72%, p<0.001) and T387 (70%, p<0.001) GICs. (E) Staining for stemness marker Nestin (yellow) with nuclei were counterstained with DAPI (cyan). Scale bar = 10μm. (F) Nestin positive cell were significantly reduced in T3691 GICs at 48h (71.2%, p<0.001) but not in (F) T387 GICs. Number of Nestin positive cells was normalized to untreated controls. Data from three experiments were normalized to liposome treated controls (a, b, d). Percent values are relative to control siRNA.

Having hypothesized that FTH1 loss would be detrimental to GICs, we measured extracellular LDH activity after knockdown. While FTH1 knockdown was associated with significantly elevated LDH levels in both subtypes (Fig 3A), T3691 cells exhibited a much higher increase compared to T387 GICs. Since FTH1 is known to have an anti-apoptotic role [24, 27], we assayed for executioner caspase 3/7 activity and found significant induction of caspase activity upon FTH1 knockdown in both GICs (Fig 3B). Further, we assessed mitochondrial damage in these cells since these organelles are key regulators of cell death [28]. Indeed we found perinuclear localization of outer membrane protein, translocase of outer mitochondrial membrane 20 (TOMM20) in both GICs (Fig 3C), typical of damaged mitochondria, while control cells were found to have an even cytosolic distribution. Western blot analysis of TOMM20 revealed significant reduction in mitochondrial mass suggesting that FTH1 knockdown resulted in mitochondrial degradation (Fig 3D and 3E). Since loss of stemness in GICs has been shown to result in differentiation and increased vulnerability to therapeutics [29], we evaluated expression of the class IV intermediate filament protein, Nestin, known to be associated with stemness and self-renewal in both GIC subtypes [30, 31]. Increase in Nestin expression has been correlated with increasing glioma grade [32] while loss of Nestin expression has been shown to impair cell growth, stem cell marker expression and impair tumorigenic ability [33, 34]. When GICs were exposed to siRNA FTH1, we found a significant loss in Nestin expression in T3691 but not T387 GICs (Fig 3F) implying that T3691 GICs might be more sensitized following FTH1 loss.

### GICs from proneural glioblastoma exhibit further reduction in cell viability upon radiation exposure

**Fig 4:**
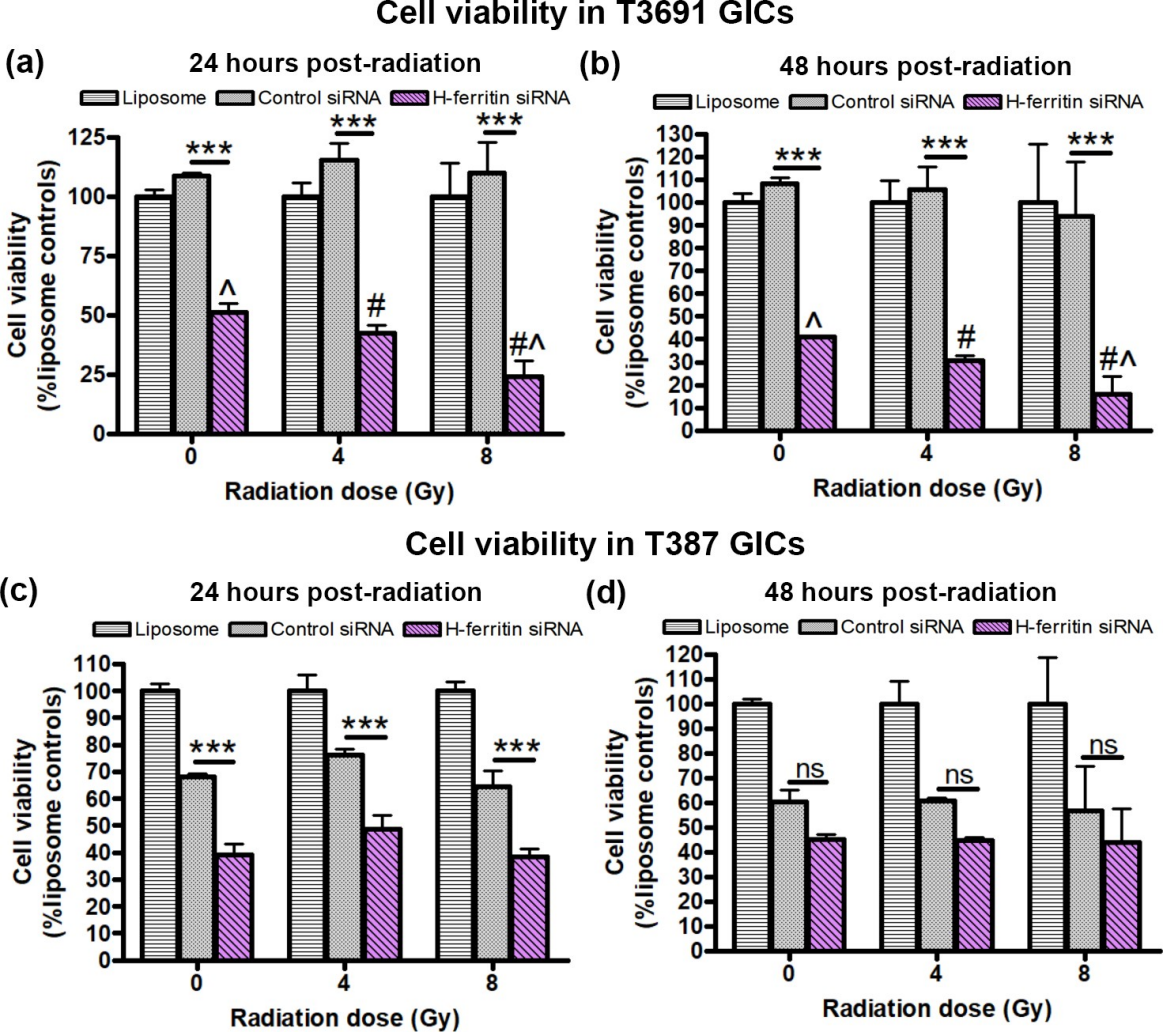
FTH1 knockdown lowers cell viability of T3691 but not T387 GICs following radiation. GICs were transfected with FTH1 or control siRNA at siRNA (μg): liposome (μl) ratios of 2:4 (T3691) or 2:8 (T387) for 24h, followed by no radiation (0Gy) or radiation at 4Gy and 8Gy. Cell viability was assessed 24 and 48 hours after radiation exposure using MTS assay. (A, B) In T3691 GICs, at both 24h (a) and 48h (b) post radiation, cell viability in FTH1 siRNA treated group was significantly reduced relative to control siRNA irrespective of radiation at 0Gy, 4Gy and 8Gy (*** = p<0.001). FTH1 siRNA treated T3691 cells radiated at 8Gy showed an additive decrease in cell viability relative to un-irradiated controls (0Gy) (Λ = p<0.01) as well as cells radiated at 4Gy (Λ # = p<0.05). However, there was no significant reduction in viability at 4Gy relative to un-irradiated controls (0Gy). (C) 24h post radiation, T387 GICs also showed significant decrease in cell viability at all three doses (*** = p<0.001). (D) T387 GICs showed no decrease in cell viability at 48h post radiation (ns = not significant). T387 GICs showed no significant change in cell viability after radiation exposure relative to un-irradiated controls (ns = not significant). Data from three experiments was normalized to liposome treated controls.

We next tested the hypothesis that FTH1 downregulation was associated with increased radiosensitivity in GICs. T3691 cells displayed a significant reduction in viability following transfection alone (0Gy, 47%) which was exacerbated by radiation exposure at 4Gy (36%) and 8Gy (22%) (Fig 4A, 24h post radiation). The same pattern was seen 48 hours post-radiation (Fig 4B). Similar to the T3691 cells, T387 cells showed a significant loss of cell viability following transfection with FTH1 siRNA at 0Gy (57%) (Fig 4C), but 24h post radiation exposure at 4Gy (64%) and 8Gy (60%) there was no further decrease in cell viability (Fig 4C). By 48h there was no significant difference in cell viability relative to control siRNA (Fig 4D).

### FTH1 is important for DNA repair and clonogenic survival in T3691 tumor derived GICs

**Fig 5:**
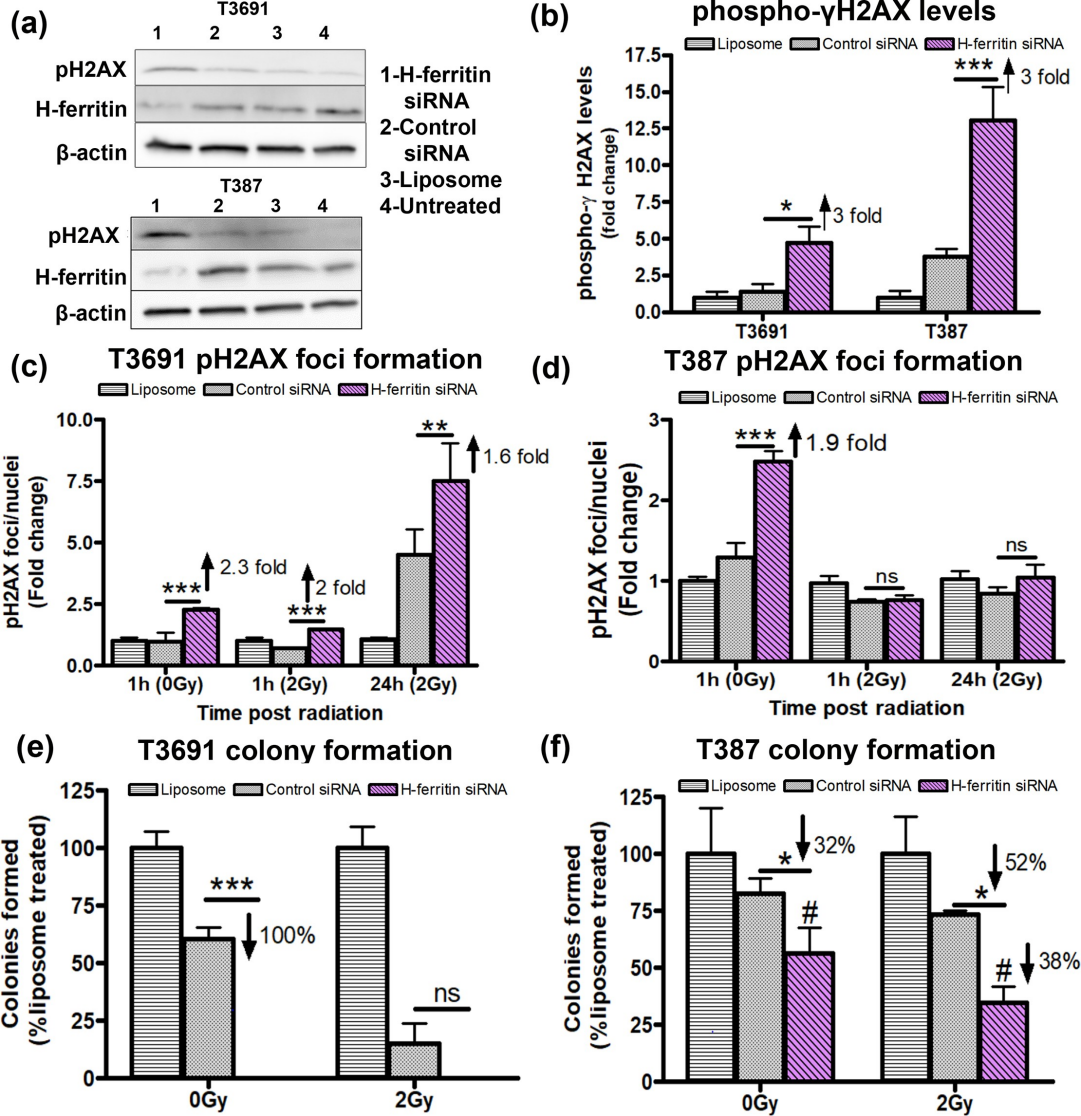
Loss of FTH1 impairs DNA repair and colony formation ability of GICs following radiation. (A) Total phospho-ϒH2AX levels were evaluated by western blot at 48h post-transfection with FTH1 siRNA. (B) Quantification of phospho-ϒH2AX levels in T3691 and T387 cells showed a 3 fold increase in phosphorylation of ϒH2AX relative to control siRNA treated cells. (C) phospho-ϒH2AX foci formation in T3691 was assessed 24h post transfection (1hr, 0Gy), 1hr and 24h post radiation at 2Gy. Compared to control siRNA, T3691 GICs showed a 2.3 fold increase in number of foci/nuclei (***p<0.001) in the absence of radiation and further continued to retain foci starting at 1hr (2 fold, ***p<0.001) and upto 24h post radiation (1.6 fold, **p<0.01). (D) T387 cells treated similarly, also showed a 1.9 fold increase in foci number (***p<0.001) at 0Gy, but showed no significant change relative to controls siRNA treated cells upon radiation. (E) T3691 GICs failed to form colonies and showed significant reduction in colony formation compared to control siRNA treated GICs (100%, ***p<0.001) in the absence of radiation. (F) T387 GICs showed a comparatively moderate decrease in colony formation without radiation at 0gy (32%, p<*0.05). Colony forming ability of FTH1 siRNA silenced GICs was further impaired by radiation (52%,*p<0.05) compared to control siRNA and compared to its un-irradiated counterpart (38%, #p<0.05). Data from three experiments was normalized to liposome treated controls.

Radiation resistance of GICs has been linked to efficient DNA repair [2]. Hence, we determined if FTH1 loss affected DNA repair ability of GICs differentially. First we assessed the levels of phospho-γH2AX protein 48h after knockdown and found similar levels of phosphorylation in both GICs (Fig 5A and 5B). Since phospho-γH2AX foci formed after radiation exposure represent double stranded DNA breaks [35, 36], we quantified the number of foci formed before radiation as well as immediately following (1h) and 24h after radiation at 2Gy. In the absence of radiation, we found comparable increase in the number of foci/nucleus in both GICs. The ratio nearly doubled by 24h after 2Gy exposure in the T3691 cells (Fig 5C) but there was no difference between groups in the T387 cells following radiation (Fig 5D). Clonogenic assay is considered an excellent monitor of cell reproductive ability. Therefore, we also measured the ability of transfected cells to form colonies *in vitro* following radiation exposure. T3691 GICs treated with FTH1 siRNA failed to form any colonies even without radiation exposure (Fig 5E). On the other hand, T387 GICs showed a moderate reduction in colony formation after FTH1 siRNA treatment that was further decreased upon radiation exposure (Fig 5F).

## Discussion

Lipid based delivery systems have the advantage of being highly adaptable since minor changes in composition allow significant alterations in their capabilities. We have previously established a liposomal formulation that can carry contrast agents to the brain [37]. However, to effectively carry siRNAs to GICs, we modified our existing formulation through addition of MVL5, a pentavalent cationic lipid. MVL5 has significant advantages over univalent lipids since they show co-operative binding at the atomic level, yielding highly efficient knockdowns with reduced toxicity and off target effects [38]. Thus, addition of MVL5 allowed us to produce lipoplexes capable of transfecting GICs.

Moreover, the need to alter siRNA: liposome ratio to produce comparable knockdown in T387 GICs highlights both the adaptability of liposomes as well as inherent differences in transfectability within GIC subtypes. There is evidence to suggest that cancer cell lines have differences in cell membrane composition [39] and that cancer initiating cells (CICs) may have unique lipid compositions that maintain their stemness [40]. Considering that these GICs come from different patients, it is possible that there are intrinsic differences in membrane composition between the two cell lines that account for lower uptake of anionic complexes in T387 cells. In an *in vivo* setting, one would provide multiple treatments of the complexes, which would negate the minor differences in knockdown seen in an *in vitro* setting. Thus, the different siRNA: liposome ratios needed to achieve comparable knockdown *in vitro* may not necessarily translate to variable levels of knockdown *in vivo*.

Another caveat of increasing liposome: siRNA ratio of complexes used for T387 transfection was the increase in cytotoxicity observed in control siRNA transfected cells. While cationic particles are more readily taken up, they also cause damage to cell membranes and result in increased cytotoxicity. We believe the net cationic charge on control siRNA: liposome complexes attributed to an increased cell membrane damage (LDH) and therefore lowered cell viability in T387 GICs. Additionally, we also observed a mild reduction in FTH1 expression in control siRNA T387 cells. Given that firefly luciferase has no mammalian counterpart, we are unsure as to why it led to the decrease of FTH1 expression in T387 cells especially since the same concentration of siRNA had no effect in T3691 GICs. However, despite this, FTH1 siRNA transfected cells show significantly higher cytotoxicity in all our assays. We also observed low colony forming ability in control siRNA transfected T3691 cells after radiation. However, given that FTH1 siRNA transfected cells were unable to form colonies even in the absence of radiation, this reduction is inconsequential.

While cancer cells utilize iron to drive tumorigenesis through increased proliferation, CICs direct the iron towards maintaining stemness [41]. This is recapitulated in GICs which are more iron-dependent than their non-stem counterparts [14]. Indeed, unlike astrocytoma cells [24], we have shown that GICs have increased sensitivity to FTH1 knockdown even in the absence of radiation implying that oxidative damage resulting from decreased FTH1 is sufficient to overwhelm the robust antioxidant systems possessed by these cells [42]. Interestingly, the increase in extracellular LDH activity far exceeded caspase 3/7 activity especially in T3691 cells. Ferroptosis is a caspase independent form of cell death characterized by increased reactive oxygen species generation and consequent lipid peroxidation as well as protein carbonylation [43, 44]. Studies have also shown that the ferroptosis inducer erastin [45] triggers LDH release as a result of lipid. In our previous studies we have shown both increase in labile iron pool as well as oxidative damage to proteins in response to FTH1 knockdown [24]. Together, this suggests that knockdown of FTH1 may trigger ferroptosis in GICs. Moreover, we observed significant damage to mitochondria in our study which could explain caspase 3/7 initiated cell death. Thus, FTH1 downregulation could be activating caspase dependent and independent cell death pathways in GICs. Moreover, since increased mitochondrial function in CICs has been linked to treatment resistance, tumorigenesis and maintenance of stemness [46] this further emphasizes the importance of FTH1 in governing multiple aspects of GIC function.

As stated previously, nuclear ferritin, comprised largely of FTH1, plays a role in DNA protection [19, 20]. Both T3691 and T387 GICs showed elevated phosphorylation of DNA damage response protein ϒH2AX following treatment with FTH1 siRNA. We did not assess levels of nuclear ferritin post knockdown but have shown previously that knockdown of FTH1 results in reduced levels of both cytoplasmic and nuclear ferritin [47]. Moreover, there was an increase in markers of radiation sensitivity in T3691 cells at relatively lower doses of radiation compared to astrocytomas cells [24]. Ataxia telangiectasia mutated (ATM), a cell-cycle checkpoint kinase is responsible for downstream activation and phosphorylation of ϒH2AX at Serine139 [48]. Unlike astrocytoma cells that showed impairment of ATM phosphorylation and therefore were presumably unable to activate ϒH2AX, we found increased levels of phospho-ϒH2AX in GICs after FTH1 knockdown. However, the ability to repair DNA, indicated by disappearance of ϒH2AX foci over time was altered in T3691 GICs implying that a downstream effector of DNA repair was disrupted following FTH1 loss in T3691 but not T387 GICs. A lack of colony formation in FTH1 knockdown T3691 cells even in the absence of radiation highlights the importance of this protein for their survival. On the other hand, a moderate impairment in colony forming ability of T387 GICs suggest that while they may be able to show efficient DNA repair in the short term, their ability to progress through cell cycle is impaired over time following FTH1 loss.

Several groups have recently illustrated that GICs with a PN signature, commonly located in perivascular regions, were more radiosensitive while MES GICs, adapted to inner hypoxic core, were highly radiation resistant [49–51]. Additionally, PN GICs are more sensitive to inhibition of expression of enhancer of zest homolog 2 (EZH2), a member of the polycomb repressive complex 2 (PRC2) while MES GICs are reliant on BMI1 (part of PRC1 complex) for maintaining their resistance and stemness [49]. Interestingly, the mitotic kinase, maternal embryonic leucine-zipper kinase (MELK), through its interaction with forkhead box protein M1 (FOXM1), has been shown to not only regulate GIC proliferation [52] but also mediate radioresistance by transcriptionally upregulating EZH2 [53]. A previous study has showed that signal transducer and activator of transcription 3 (STAT3), an important CIC node that is aberrantly activated in glioblastoma [54], is dependent on ferritin expression for its activation and in turn regulates GIC self-renewal and tumorigenicity through downstream activation of FOXM1 [14]. This STAT3-FOXM1 pathway was found to be sensitive to changes in iron homeostasis, with ferritin knockdown leading to reduced activation of both proteins. Moreover, STAT3-FOXM1 interaction has been shown to mediate radioresistance in GICs [55], further lending credence to downregulation of FTH1 as a viable target for radiosensitization in these cells. Furthermore, EZH2 is also a known regulator of Nestin expression in malignant gliomas [56], which we found to be decreased in PN but not MES GICs after FTH1 knockdown in our study. Together, these data suggest that disruption of a STAT3-FOXM1-EZH2 axis, through silencing of FTH1, might be responsible for increased sensitivity of PN GICs following FTH1 downregulation and underscores the differential mechanisms that govern the PN and MES subtypes of GICs.

In summary, we show that modulating the expression of a single gene can alter the molecular profile of glioma initiating cells but we also show that the GICs subtypes are differentially sensitive to FTH1 knockdown. Although using antisense RNA technology to target genes governing DNA repair [57, 58] and drug resistance [59, 60] has proven to be effective at improving radiation and chemotherapeutic sensitivity respectively, these studies rely on delivering siRNA through commercially available transfection reagents that preclude clinical use. Liposomes, due to their well-known ability to encapsulate drugs and nucleic acids, pliability to surface modifications and superior performance *in vivo*, are increasingly being used to target CICs [61]. Our study demonstrates that liposomes are an efficient new tool for transfection and modulation of gene expression in patient derived GICs. Furthermore, in combination with our previous study, we continue to develop evidence that FTH1 is an important factor for survival in GICs and a viable target for destruction of cancer cells, including GICs.

## Supporting information

S1 Fig Gel retardation assay to measure mobility of liposome: siRNA complexes. Gel retardation assay using both control luciferase siRNA and FTH1 siRNA complexed with MVCL in a siRNA (μg): MVCL (μl) ratio of 2:4 or 2:8 showed retention of complexes near the well indicating slower migration compared to free siRNA indicating efficient complexation.
(TIF)
