# Supplementary figures and images for: Liposomal delivery of ferritin heavy chain 1 (FTH1) siRNA in patient xenograft derived glioblastoma initiating cells suggests different sensitivities to radiation and distinct survival mechanisms
Source: PLoS One. 2019 Sep 6;14(9):e0221952. doi: 10.1371/journal.pone.0221952 (PMC6730865; doi:10.1371/journal.pone.0221952)

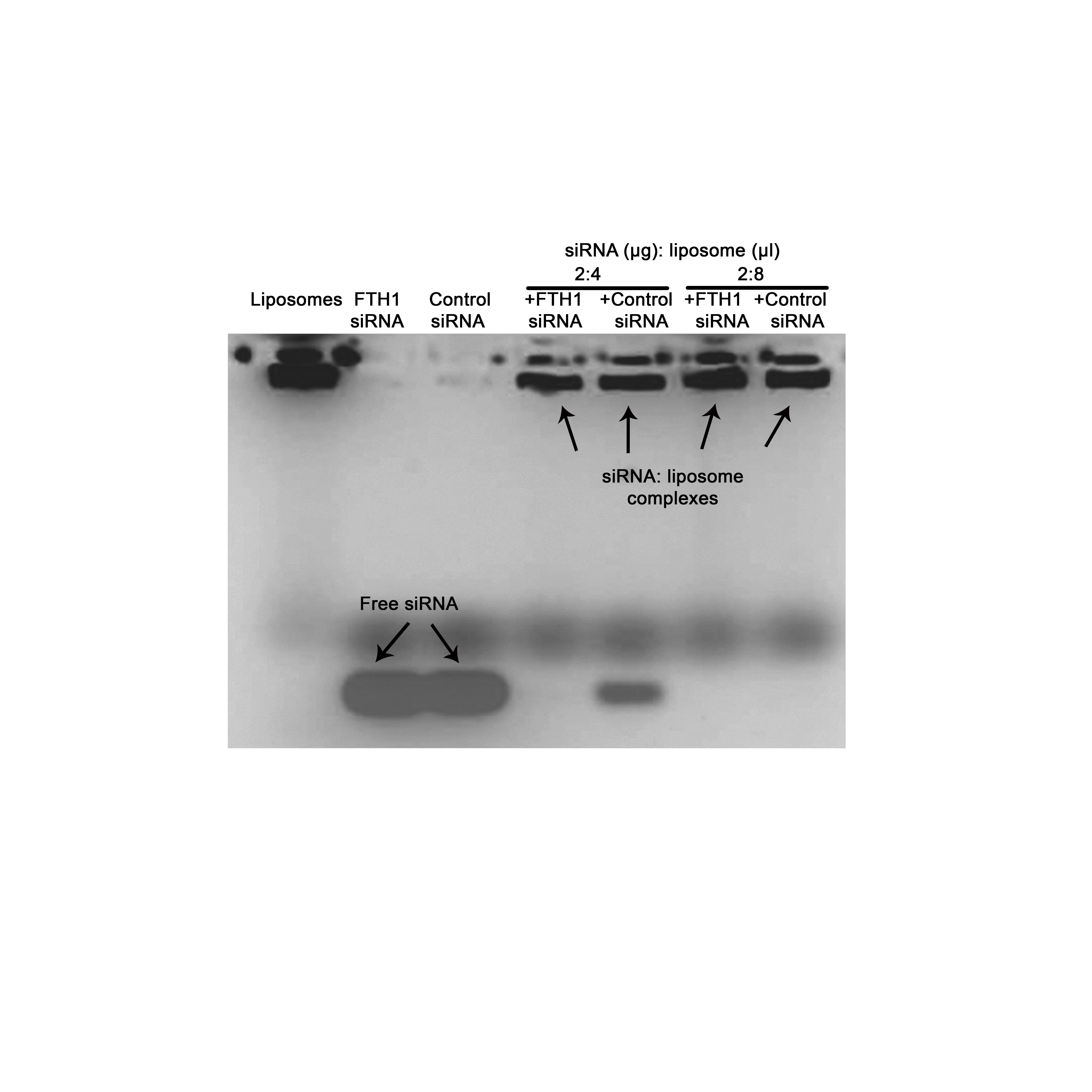

Supplement: S1 Fig — Gel retardation assay using both control luciferase siRNA and FTH1 siRNA complexed with MVCL in a siRNA (μg): MVCL (μl) ratio of 2:4 or 2:8 showed retention of complexes near the well indicating slower migration compared to free siRNA indicating efficient complexation. (TIF) [file pone.0221952.s001.tif]
